# In-plane direct current probing for spin orbit torque-driven effective fields in perpendicularly magnetized heavy metal/ferromagnet/oxide frames

**DOI:** 10.1038/s41598-018-29397-4

**Published:** 2018-07-23

**Authors:** Seungmo Yang, Jinhyung Choi, Junghoon Shin, Kapsoo Yoon, Jungyup Yang, JinPyo Hong

**Affiliations:** 1 Novel Functional Materials and Device Laboratory, Research Institute of Natural Science, Department of Physics, Hanyang University, Seoul, 133-791 Korea; 2 Department of Physics, Kunsan National University, Kunsan, South Korea

## Abstract

Electrical manipulation of magnetization states has been the subject of intense focus as it is a long-standing goal in the emerging field of spintronics. In particular, torque generated by an in-plane current with a strong spin-orbit interaction shows promise for control of the adjacent ferromagnetic state in heavy-metal/ferromagnet/oxide frames. Thus, the ability to unlock precise spin orbit torque-driven effective fields represents one of the key approaches in this work. Here, we address an in-plane direct current measurement approach as a generic alternative tool to identify spin orbit torque-driven effective fields in a full polar angle range without adopting the commonly used harmonic analyses. Our experimental results exhibited a strongly polar angular dependency of the spin orbit torque-driven effective fields observed from Ta or W/CoFeM/MgO frames.

## Introduction

Recently, intentional manipulation of magnetization dynamics through the electric fields and current continues to be of significant interest from a fundamental perspective and for spintronic technologies. In particular, the in-plane current-driven spin orbit interaction (SOI) phenomenon, which is the so called spin-orbit torque (SOT), has garnered considerable interest for controlling magnetization in the adjacent ferromagnet. Electric currents in large SOI materials can induce a non-zero spin current or spin accumulation in a direction perpendicular to the charge flow via a conversion between electron spin and linear momentum, which can exert torque on the magnetization in the adjacent ferromagnets. A variety of works have been published on the nature of the SOT-related magnetic dynamics including magnetization switching^1–4^, logic operations^5^, and domain-wall motion^6,7^ by considering two dominant mechanisms. One is the spin Hall Effect (SHE) arising from bulk materials with a large SOI (heavy metal layer)^8^, and the other is the Rashba effect, which occurs at the interface between the heavy metal (HM) layer and ferromagnetic (FM) layer^9,10^. However, while both dynamics can provide two effective magnetic torques, which are commonly called the field-like torque (${\overrightarrow{{\rm T}}}_{f} \sim {{\rm T}}_{f}\hat{M}\times \overrightarrow{\sigma }$) and the damping-like torque $\overrightarrow{({T}_{d}} \sim {{\rm T}}_{d}\hat{M}\times (\hat{M}\times \overrightarrow{\sigma }))$, the relative magnitude and direction of *T*_*f*_ and *T*_*d*_ vary strongly depending on the stack configuration, the choice of HM, and oxidation degree^1,11^. In addition, previous studies have shown that the *T*_*f*_ is mainly induced by the Rashba effect, while the *T*_*d*_ is mostly governed by the spin Hall effect^12^.

To date, numerous studies have also reported the successful determination of each effective magnetic field by means of diverse analysis approaches, such as field-current equivalence^13^ and harmonic analysis^11,12,14^. However, the experimentally observed torque components (*T*_*f*_ and *T*_*d*_) evaluated even in the same structural frames exhibited distinct measurement or analysis dependence. For instance, Chen *et al*.^15^ reported that the T_f_ is nearly three times larger than the T_d_, while Liu *et al*.^3^ presented a large SHE-induced torque in Ta/CoFeB/MgO with a perpendicular magnetic anisotropy (PMA) feature. Furthermore, the suggested two underlying physics for spin-orbit torque (that is, the Rashba effect and SHE) cannot explain several reported phenomena, such as the influence of oxygen bonding state^1^ and polar angle of magnetization^15^. Thus, the ability to identify each individual torque component with their precise magnitudes and to establish a firm understanding of their physical origins remain key steps toward extending the use of these materials in emerging spintronic applications.

A unique concept in this work is the use of an in-plane direct current (DC) source as an independent tool for the determination of two current-induced effective magnetic fields in the representative HM/CoFeB/MgO stacks without the need for the commonly used harmonic measurement technologies. Several groups have already reported DC current technique to calibrate the SOT effective fields, our work shows some uniqueness compared the previous works. M. Kawaguchi *et al*.^16^ reported two components of current-induced SOT along the y-axis (transverse field in the paper) and the z-axis (perpendicular field in the paper) for especial in-plane magnetic easy axis films. This approach might not be effective to the PMA-based frames. As is well-known, the PMA-based frames have widely been the focus of immense interest for the device applications and academic research. Second, J. Han *et al*.^17^ also addressed the quantitative calibration of SOT using DC current-based analysis in PMA-based topological insulator frames. However, this work has considered only damping-like torque in the analysis: For example, only H_z_ field was considered by employing the dominant current-induced switching along the z-axis. Thus, the uniqueness of our work serves to provide the approach for the determination of two separated spin torque components including Field-like torque and Damping-like torque in PMA-based frames.

We address the individual in-plane DC-induced SOT effective fields in both Ta and W-based HM/CoFeB/MgO stacks through control of various symmetry features. In particular, the dependence of the DC-induced effective magnetic fields on the magnetization polar angle (θ) in a full θ range was extensively evaluated, along with quantitative reliability tests afforded by the previously reported analysis approaches, including the field balance and energy equilibrium equations. An interfacial spin transparency concept that can occur at the HM/FM/oxide interface is briefly described based on the experimental findings.

## Results and Discussion

**Figure 1:**
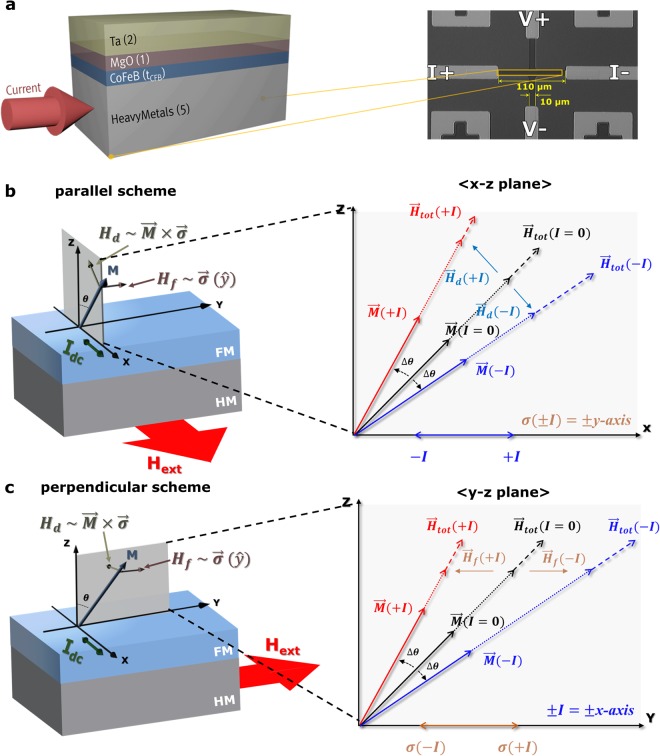
Schematics of sample stacks and individual current-induced effective fields provided by DC application. (**a**) Sample architecture (Left) and an optical image of the Hall bar device (Right). The current-induced effective field illustrations for individual SOT components (*H*_*d*_ & *H*_*f*_) in the presence of H_ext_ (**b**) parallel and (**c**) perpendicular to the direct current I_dc_ sources. Enlarged figures of (**b** and **c**) indicate significant shifts (red and blue colors) in the magnetization directions from the equilibrium state in the presence of the positive and negative DC sources, thereby ensuring a direct estimate of the individual *H*_*d*_ & *H*_*f*_ in z-x and z-y planes that depend on the parallel and perpendicular schemes, respectively.

Figure 1a shows an optical microscope image of the fabricated Hall device with a 10 μm width. A more detailed description of the fabrication process is given in the Methods section. The sample stacks used have the structure of Si/SiO_2_ (200)/HM (5)/CoFeB (1.2)/MgO (1)/Ta (3) (thicknesses in nanometers) and were post-annealed at a moderate temperature, where two different annealing temperatures are chosen for 250 °C for Ta and 350 °C for W HMs. Hereafter, the Ta-based sample is referred to as ‘Sample A,’ and the W-based sample is ‘Sample B’. All samples exhibit typical PMA features due to the presence of interface anisotropy at CoFeB/MgO that is attributed to the hybridization of Fe 3d and O 2p orbitals^18^ after the annealing process (see Supplementary Information 3). Figure 1b and c illustrate the DC source-driven SOT effective magnetic fields in the presence of an applied magnetic field (*H*_*ext*_), along with the definition of the coordinate system. The current-induced effective magnetic fields have two components: the field-like effective field (H_f_) and the damping-like effective field (H_*d*_), which are defined by ${\overrightarrow{H}}_{f}\equiv {{\rm{T}}}_{{\rm{f}}}\overrightarrow{\sigma }$, ${\overrightarrow{{\rm{H}}}}_{d}\equiv {T}_{d}(\hat{M}\times \overrightarrow{\sigma })$ from the two torque relations ${\overrightarrow{{\rm T}}}_{f}={{\rm T}}_{f}\hat{M}\times \overrightarrow{\sigma }$ and $\overrightarrow{{T}_{d}}={{\rm{{\rm T}}}}_{d}\hat{M}\times (\hat{M}\times \overrightarrow{\sigma })$. Two separate measurement schemes were introduced with the enlarged figures (right figures). One is the parallel measurement scheme (left of Fig. 1b), and the other is the perpendicular measurement scheme (left of Fig. 1c). In both schemes, the *H*_*ext*_ with a tilting angle θ = 85° from vertical is swept in a wide range to maintain a single domain state during magnetization switching. In the parallel scheme, DC current (*I*_*dc*_) with a magnitude of ±0.5 mA was injected along the x-axis and *H*_*ext*_ were applied in the x-z plane with θ_H_ = 85°, while the *H*_*ext*_ was applied in the y-z plane with θ_H_ = 85° with the current flow along the x-axis for the perpendicular scheme. The enlarged figures (rights) of Fig. 1b and c also depict the key concept of magnetization tilting upon application of positive and negative ±*I*_*dc*_ in both schemes. For zero current (I_dc_ = 0), the direction of a magnetization vector ($\overrightarrow{{\rm{M}}}$) in an FM layer is placed at the equilibrium position (θ_0_, *φ*_0_) and is parallel to the sum of only two magnetic fields: ${\overrightarrow{{\rm{H}}}}_{0}(\equiv {\overrightarrow{H}}_{an}+{\overrightarrow{H}}_{ext})$, where the anisotropic field (${\overrightarrow{{\rm{H}}}}_{an}$) and external field (..). Both magnetic parameters can be determined experimentally. When the I_dc_ is applied through either an HM layer or an FM layer, the current-induced torque will allow for magnetization tilting of the FM layer toward the new equilibrium position (θ, *φ*) from the equilibrium position (θ_0_, *φ*_0_). That is, an I_dc_ sign with a finite amplitude leads to relative magnetization tilting with an amplitude of ${\rm{\Delta }}{\rm{\theta }}$ from the equilibrium state. Note that, in this analysis, the variations in the polar and azimuthal angles of magnetizations (${\rm{\Delta }}{\rm{\theta }},\,{\rm{\Delta }}{\rm{\phi }}$) mainly originate from the SOTs induced by the application of I_dc_ because of the relatively small magnitude of the current-generated Oersted magnetic field (0.314 Oe, See Supplementary Information 9). In this regard, an I_dc_-induced variation in magnetization establishes the experimental difference in the total Hall signal (R_H_) observed in both schemes. Thus, tailoring the ${\rm{\Delta }}{\rm{\theta }}\,{\rm{and}}\,{\rm{\Delta }}{\rm{\phi }}$ upon application of I_dc_ is a crucial step for determining the individual current-induced effective magnetic fields. Here, two measurement schemes are chosen to separately determine each individual ${\overrightarrow{{\rm{H}}}}_{f}$ and ${\overrightarrow{H}}_{d}$. In the two schemes, the injection of an identical current (x-axis) is supposed to create the same direction of ${\overrightarrow{{\rm{H}}}}_{f}$ (always along the y-axis) with the same magnitude due to the presence of the same spin polarization ($\overrightarrow{{\rm{\sigma }}}$), which is one parameter for the exchange interaction with the magnetization ($\overrightarrow{{\rm{M}}}$) under the Rashba effect-related torque. That is, the ${\overrightarrow{{\rm{H}}}}_{f}$ ${(\overrightarrow{{\rm{H}}}}_{f} \sim \overrightarrow{\sigma })$ is independent of the direction of magnetization. However, as the ${\overrightarrow{H}}_{d}$ may arise mainly from the spin current created by the SHE through the spin transfer torque (STT) phenomenon, the ${\overrightarrow{H}}_{d}$ (${\overrightarrow{{\rm{H}}}}_{d} \sim \overrightarrow{M}\times \overrightarrow{\sigma }$) relies on the initial magnetization direction. Thus, our work is hereafter discussed based on the simplest assumption in which the ${\rm{\Delta }}{\rm{\theta }}$ in a parallel scheme is only given by the ${\overrightarrow{{\rm{H}}}}_{d}$ in the x-z plane, and the ${\rm{\Delta }}{\rm{\theta }}$ in a perpendicular scheme is only governed by the ${\overrightarrow{H}}_{f}$ in the y-z plane. That is, the separate determination of ${\rm{\Delta }}{\rm{\theta }}$ in the two schemes serves to determine the strengths of each ${\overrightarrow{{\rm{H}}}}_{f}$ and ${\overrightarrow{H}}_{d}$. This is a key concept in the application of ±*I*_*dc*_ without adopting the commonly used harmonic analyses.

**Figure 2:**
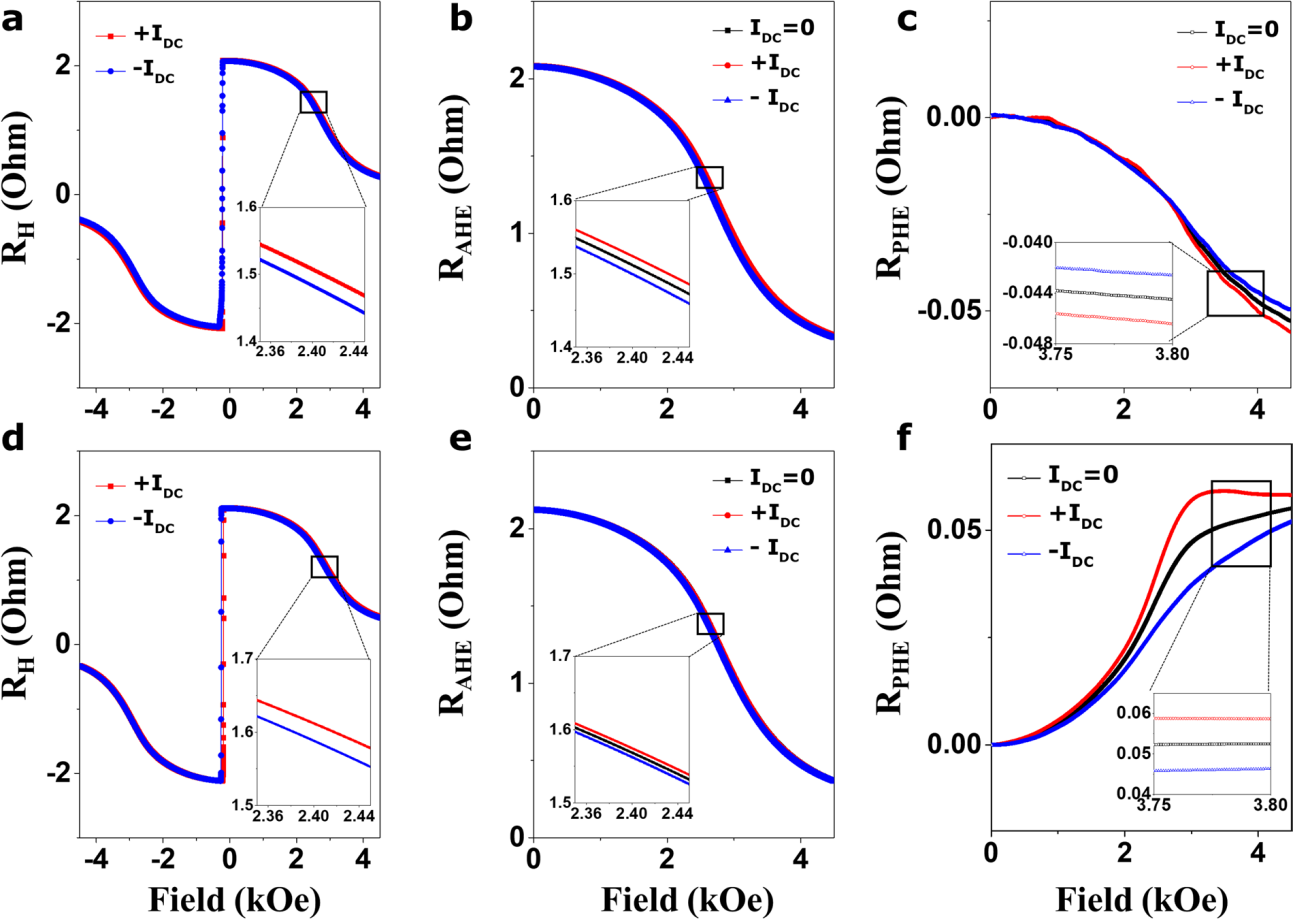
Magnetic features of the Ta-based sample [Sample A] under a DC source for two measurement schemes. (**a**) Hall resistance *R*_*H*_ versus H_ext_ parallel to the positive (red line) and negative (blue line) current I_dc_ with a magnitude of 0.5 mA. (**b**,**c**) Separated anomalous Hall effect signal (*R*_*AHE*_) and planar Hall effect signal (R_PHE_) from the *R*_*H*_ as a function of H_ext_ parallel to zero-current (black line), +0.5 mA (red line) and −0.5 mA (blue line). (**d**) Hall resistance *R*_*H*_ verse H_ext_ perpendicular to the positive (red line) and negative (blue line) current I_dc_ with a magnitude of 0.5 mA. Similarly, (**e**,**f**) *R*_*AHE*_ and R_PHE_ versus H_ext_ perpendicular to zero-current (black line), +0.5 mA (red line) and −0.5 mA (blue line). Insets: the magnified features of the graphs reflect the obvious difference in the main curves recorded at ±*I*_*dc*_ current, which lowered the magnetization in one direction and increased it in the other direction.

Considering the above concept, Fig. 2a and d show plots of the representative Hall resistance signal R_H_ of Sample A as a function of ${\overrightarrow{{\rm{H}}}}_{ext}$ upon the same magnitude of positive (red) and negative (blue color) *I*_*dc*_ for both parallel (Fig. 2a) and perpendicular (Fig. 2d) schemes, respectively, where the magnitude of the applied *I*_*dc*_ is ±0.5 mA. As depicted above, the insets of Fig. 2a,d clearly show the distinct dependence of R_H_ on the direction of *I*_*dc*_; that is, R_H_ increased for a positive *I*_*dc*_ (red color) and decreased for a negative *I*_*dc*_ (blue color). Thus, to identify the observed R_H_ by the application of *I*_*dc*_, three possible contributions to the R_H_ curve were assumed as a first step as follows:

1 $${R}_{H}({I}_{dc})={R}_{OHE}({I}_{dc})+{R}_{AHE}\cdot \,\cos \,\theta ({I}_{dc})+{R}_{PHE}\cdot {\sin }^{2}\theta ({I}_{dc})\cdot \,\sin \,2\phi ({I}_{dc})$$

where θ is the polar angle, *φ* is the azimuthal angle of the magnetization, and R_AHE_ and R_PHE_ are the anomalous Hall effect (AHE) and planar Hall effect (PHE) resistances, respectively. Note that a linear background (first term of Equation (1)) generated from the ordinary Hall effect (*R*_*OHE*_) is excluded due to its negligible magnitude in this analysis. To separate the total observed R_H_ into a pristine Hall signal (R_H0_) upon zero current and a variation in the Hall resistance (${{\rm{\Delta }}R}_{{\rm{H}}}$) upon the injection of *I*_*dc*_ with a certain magnitude, the R_H_ of Equation (1) was approximated using a Taylor expansion only to the first order as follows:

2 $${R}_{H}({I}_{dc})\cong {R}_{H}({I}_{dc}=0)+{I}_{dc}\cdot {\frac{d{R}_{H}}{dI}}_{I=0}\equiv {R}_{H0}+{\rm{\Delta }}{R}_{H},$$

where the Hall resistance in the absence of *I*_*dc*_ is denoted by ${{\rm{R}}}_{{\rm{H}}0}\equiv {{\rm{R}}}_{{\rm{H}}}({I}_{dc}=0)$, and the variation of R_H_ by the I_DC_ is ΔR_H_. The R_H0_ and ${{\rm{\Delta }}R}_{{\rm{H}}}$ upon ±*I*_*dc*_ can be obtained by

3 $${{\rm{R}}}_{{\rm{H}}0}=\frac{{R}_{H}({I}_{dc})+{R}_{H}(\,-\,{I}_{dc})}{2},{\rm{\Delta }}{R}_{H}=\frac{{R}_{H}({I}_{dc})-{R}_{H}(\,-\,{I}_{dc})}{2}$$

Similarly, the two AHE- and PHE-associated terms with respect to ±H_ext_ in the presence of *I*_*dc*_ can be determined by the resulting expressions:

4 $${{\rm{R}}}_{{\rm{AHE}}}\cdot \,\cos \,\theta =\frac{{R}_{H}({H}_{ext})-{R}_{H}(\,-\,{H}_{ext})}{2},{R}_{PHE}\cdot {\sin }^{2}\theta \cdot \,\sin \,2\phi =\frac{{R}_{H}({H}_{ext})+{R}_{H}(\,-\,{H}_{ext})}{2},$$

where detailed equations are given in Supplementary Information 1. Use of the above relations enables the determination of contributions to the total R_H_. Figure 2b,c,e, and f plot the separated *R*_*AHE*_ and *R*_*PHE*_ signals of Sample A at three different *I*_*dc*_ injections (*I*_*dc*_ = +0.5 mA, 0, −0.5 mA) for both parallel (Fig. 2b,c) and perpendicular (Fig. 2e,f) schemes, where the plots are given only in the positive ${\overrightarrow{{\rm{H}}}}_{ext}$ region due to the symmetric features in the R_H_ curves of Fig. 2a,d. The separated *R*_*AHE*_ and *R*_*PHE*_ at the zero current (I = 0) are obtained by the R_H0_ expression in equation (3). The insets of Fig. 2b,e show that reversing the sign of *I*_*dc*_ clearly leads to the up (red line) and down (blue line) *R*_*AHE*_ curves of Sample A, along with the *R*_*AHE*_ (black line) upon zero current. The insets of Fig. 2c,f show similar trends for the *R*_*PHE*_ curves that are dependent on the sign of *I*_*dc*_. In addition, a closer investigation of Fig. 2b,c suggests that *R*_*PHE*_ is small (i.e., a factor of 10^2^ and 10 less than values of *R*_*AHE*_ for Samples A and B, respectively), reflecting a slight contribution to the rotational variance around the *φ* upon *I*_*dc*_ application. Thus, the magnitude of *R*_*PHE*_ can be used to determine current-induced effective fields only by means of the ${\rm{\Delta }}{R}_{AHE}$ estimated for both schemes because the contribution of *R*_*PHE*_ to the *R*_*H*_ is negligible in our work. Similarly, the R_H_, *R*_*AHE*_, and *R*_*PHE*_ trends of Sample B are similar to the observations of Sample A. More detailed plots for Sample B are presented in Fig. S2 of the Supporting Information. We believe that the determined *R*_*AHE*_ and *R*_*PHE*_ are primarily the consequence of magnetization tilting induced by the application of *I*_*dc*_, as depicted in Fig. 1b,c.

**Figure 3:**
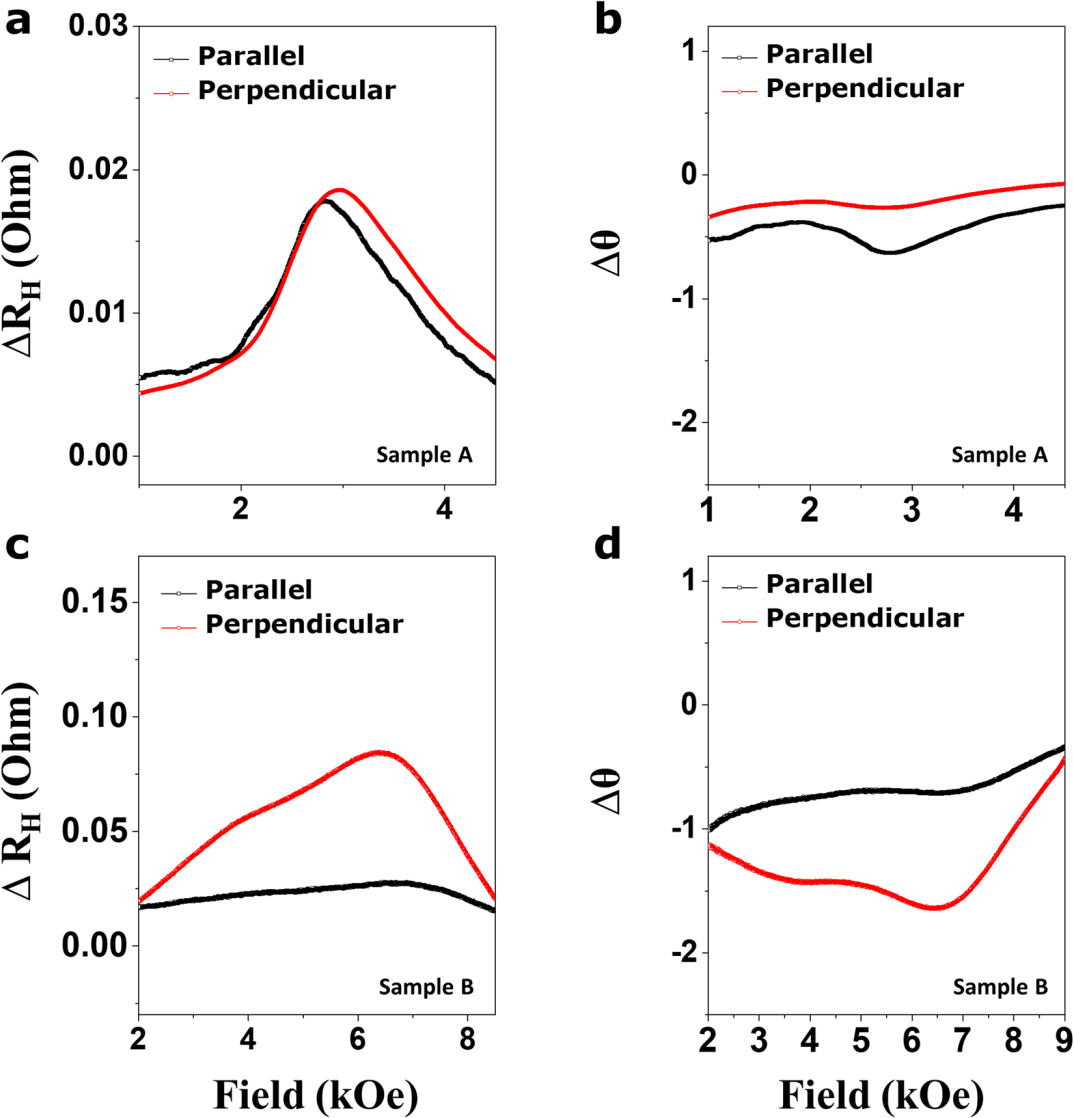
Variations in Hall resistance and polar angles for Samples A and B. (**a**,**b**) Plots of Hall resistance variation ${{\rm{\Delta }}R}_{{\rm{H}}}$ and polar angle change ${\rm{\Delta }}{\rm{\theta }}$ versus H_ext_ parallel (black line) and perpendicular (red line) to the current direction for Sample A. (**c**,**d**) Similar plots of ${{\rm{\Delta }}R}_{{\rm{H}}}$ and ${\rm{\Delta }}{\rm{\theta }}$ versus H_ext_ parallel (black line) and perpendicular (red line) to the current direction for Sample B, where a current of 0.5 mA was applied for both samples.

Figure 3a,c show the variation in Hall resistance (${{\rm{\Delta }}R}_{{\rm{H}}}$) for Samples A and B as a function of H_ext_ in both schemes. Both samples reveal the strong dependence of ${{\rm{\Delta }}R}_{{\rm{H}}}$ on H_ext_. As is evident in this figure, Sample B yields larger ${{\rm{\Delta }}R}_{{\rm{H}}}$ and ${\rm{\Delta }}{\rm{\theta }}$ than those of Sample A, possibly reflecting the presence of the large SOT initially created by the W buffer layer during growth or annealing compared to the Ta buffer layer. The possible presence of larger SOTs in Sample B (W-based frame) could be linked to both the large spin Hall angle (SHA) that is initially present in a highly resistive β-phase W layer or the extrinsic SHE nature of the sample. To illustrate the SHA contribution to the larger SOT, numerous studies have addressed the presence of the extraordinary large SHA in the β-phase W (${{\rm{\theta }}}_{{\rm{SH}}}^{\beta -W}$), which was 2 times^19^ or almost 4 times^20^ larger than those in the β-phase Ta (${{\rm{\theta }}}_{{\rm{SH}}}^{\beta -Ta}$). In addition, Demasius *et al*.^21^ have also reported the enhancement of SOT by controlling the oxygen concentration in the β-W film. These works suggest the possibility of a large SHA value in β-W by considering the special microstructure (A15) of β-W, while the α-phase W with a BCC structure is expected to have a smaller SHA value. Thus, the use of the β-phase W buffer layer in Sample B corresponds to the presence of a large SOT in our work (see the XRD analysis given in Supplementary Information 4 and the determined resistivity 172 μΩ∙cm, 139 μΩ∙cm for Ta and W, respectively). Secondly, it is widely believed that three main mechanisms have a significant impact on the SHE dynamics: an intrinsic mechanism, skew scattering, and side-jump scattering. The last two scattering terms are the so-called extrinsic mechanism, where the scattering sources are likely to arise from impurities or defects generated mainly in the HMs. In general, post annealing is a generic approach for the formation of well-aligned crystalline regions through boron out-diffusion from the CoFeB FM layer. This process ensures that the interface PMA features operate reliably to meet the demand of PMA-based devices. Thus, thermal annealing of Samples A and B lets thermally-activated B ions diffuse toward the HM layer or thermally-activated Ta or W ions migrate toward the FM layer, causing atomic intermixing of the layers^22^. According to recent work^23^, a heavy metal dopant on the ferromagnet resulted in an enhancement in SOI. In this regard, annealing results in suitable atomic mixing with Co, Fe, B, HM, or oxygen atoms in an FM or HM layer, reflecting the presence of an enhanced SOI-driven SHA in Sample B. Third, the different crystalline states observed within the two Ta and W buffer layers are also related to the SOI features. The Ta buffer layer exhibits a nearly β-phase structure, while the W buffer layer reveals a A15 crystalline structure after annealing (See XRD analysis given in Supplementary Information 4 and the determined resistivity of 172 μΩ∙cm, 139 μΩ∙cm for Ta and W, respectively). Therefore, HM and FM can include different defects or impurities after annealing. Finally, the affinity of B for the Ta and W buffer layers can likely help reduce the amount of defects or impurities within the HMs since B diffusion is different even at the same annealing temperature^24^. Figure 3a,c also show that a relatively large ${{\rm{\Delta }}R}_{{\rm{H}}}$ occurs at around 3 kOe for Sample A and 6 kOe for Sample B, in which fields of 3 and 6 kOe are very close to the anisotropy fields (H_k_) of Samples A and B, respectively (see Supplementary Information 3). Similarly, Fig. 3b,d show the change in magnetization polar angle (${\rm{\Delta }}{\rm{\theta }}$) induced by the positive current (+0.5 mA) injection, which is determined from the two separated AHE signals (I = 0, +0.5 mA) for Samples A and B. A relatively large ${\rm{\Delta }}{\rm{\theta }}$ in both samples also becomes obvious in the vicinity of the ${{\rm{H}}}_{{\rm{k}}}s$. Thus, the similarities in peaks of ${{\rm{\Delta }}R}_{{\rm{H}}}$ and ${\rm{\Delta }}{\rm{\theta }}$ in the vicinity of the ${{\rm{H}}}_{{\rm{k}}}s$ implies that their natures are similar regardless of whether the Ta or W buffer layer is used. However, the peak behavior in the vicinity of the ${{\rm{H}}}_{{\rm{k}}}s$ for Samples A and B is not clearly understood at present.

Differences in ${\rm{\Delta }}{\rm{\theta }}$s are commonly caused by variations in the magnitudes of H_f_ and H_d_, which are separately determined from the parallel and perpendicular schemes. As displayed in Fig. 3b,d, the H_ext_-dependency of ${\rm{\Delta }}{\rm{\theta }}$ implies that the magnitude of the current-induced torques is not constant with regard to H_ext_ or θ. In this sense, the specific dependence of θ on the magnitudes of H_f_ and H_d_ can be obtained from the observed ${\rm{\Delta }}{\rm{\theta }}$ traces to further explore the nature for the SOTs in the FM layer. Thus, to derive the magnitude of each H_f_ and H_d_ from the observed θ, the total magnetic energy of a perpendicularly magnetized system with the injected current can be expressed as

5 $${\rm{E}}=-{{\rm{K}}}_{{\rm{eff}}}\,{\cos }^{2}\theta -\overrightarrow{M}\cdot {\overrightarrow{H}}_{tot}$$

Here, ${\overrightarrow{{\rm{H}}}}_{tot}\equiv {\overrightarrow{H}}_{ext}+{\overrightarrow{H}}_{f}+{\overrightarrow{H}}_{d}$, where ${\overrightarrow{H}}_{f}\equiv {H}_{{\rm{f}}}\overrightarrow{\sigma }$, ${\overrightarrow{{\rm{H}}}}_{d}\equiv {H}_{d}(\hat{M}\times \overrightarrow{\sigma })$, ${\overrightarrow{{\rm{H}}}}_{ext}$ is an external magnetic field, $\overrightarrow{{\rm{\sigma }}}$ is the spin direction of spin-polarized electron, s and $\overrightarrow{{\rm{M}}}$ is magnetization of an FM layer. Under this condition, the H_d_ and H_f_ from each schemes are given by^15,25^

6 $${H}_{d}={\rm{\Delta }}\theta ({H}_{k}\,\cos \,2{\theta }_{0}+{H}_{ext}\,\cos ({\theta }_{H}-{\theta }_{0})$$

7 $${H}_{f}=[{\rm{\Delta }}\theta ({H}_{k}\,\cos \,2{\theta }_{0}+{H}_{ext}\,\cos ({\theta }_{H}-{\theta }_{0})]/\cos \,{\theta }_{0}.$$

where H_k_ is the anisotropy field $({{\rm{H}}}_{{\rm{k}}}\equiv \frac{2{K}_{eff}}{{M}_{s}})$. A more detailed description of the above equations is given in Supplementary Information 1.

**Figure 4:**
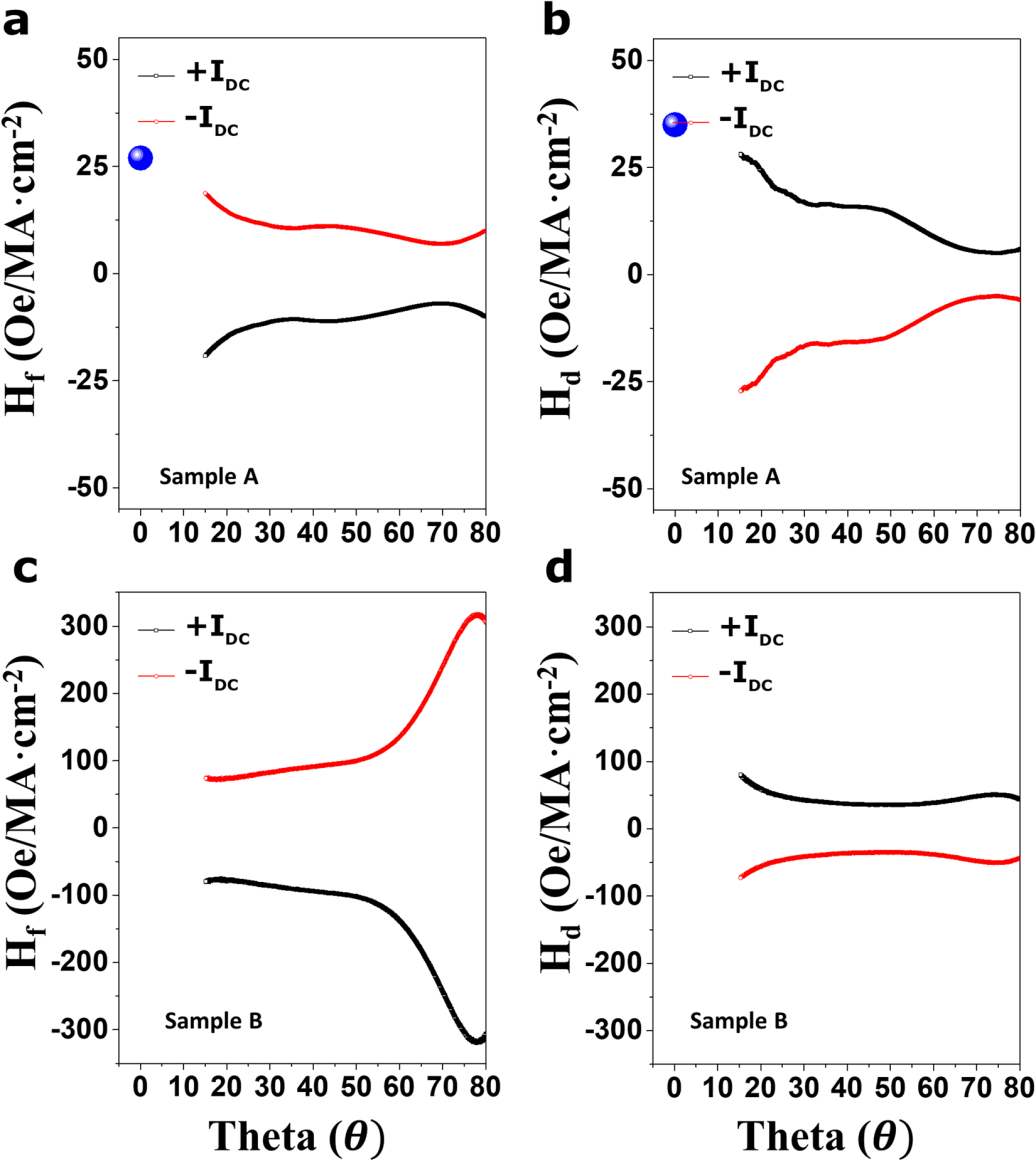
Angular dependence traces of current-driven *H*_*d*_ and *H*_*f*_ components. Each component of the current-induced effective fields was calculated from the parallel and perpendicular configurations for Samples A and B. Plots of the *H*_*f*_ components versus θ under positive (black line) and negative currents (red line) for (**a**) Sample A and (**c**) Sample B. Plots of the *H*_*d*_components versus θ for (**b**) Sample A and (**d**) Sample B. The corresponding results reflect the strong angular dependency of H_f_ and H_d_ components, where the applied magnitude of the current was 0.5 mA. Blue spheres indicate the obtained data from typical harmonic analysis.

Figure 4 exhibits the θ-dependence of H_d_ and H_f_ for Samples A (Fig. 4a,b) and B (Fig. 4c,d) under positive (black) and negative (red)*I*_*dc*_. Plots of Sample A present the typical behaviors in sign and magnitude observed from the previously reported works^11,12,26^, where the representatively reported average magnitudes of H_d_ and H_f_ for Sample A are ~20 Oe/MA∙cm^−2^, which corresponds to the obtained values from the typical harmonic analysis for Sample A (blue spheres). As harmonic signal-based analysis cannot be applied to W-based samples^27^, harmonic analysis for Sample B has not been carried out. In addition, the amplitude of H_*d*_ of Sample A is always larger than that of the H_*f*_ over the whole θ range ${(H}_{d} > {{\rm{H}}}_{f})$. This is similar to previously reported works (~2 times larger)^14^. However, such a trend is reversed in Sample B, where the amplitude of H_*f*_ is always larger than that of H_d_ in the full θ range ${(H}_{d} < {{\rm{H}}}_{{\rm{f}}})$. The dissimilarities in Samples A and B imply that their natures are also dissimilar, i.e., the SHE and Rashba contributions to the H_d_ and H_f_ are different for each material. Based on previous studies^12^, we expect two major contributions to the dissimilarities of Samples A and B, respectively. First, if the relatively large SHE for Sample B is assumed to arise from β-phase W or suitable impurities or defects, a larger SHA value of about 0.3 for Sample B allows efficient generation of a more abundant spin current $(\overrightarrow{{{\rm{J}}}_{{\rm{s}}}}={\theta }_{SH}\cdot \frac{\hslash }{2e}\cdot \overrightarrow{{J}_{c}}\times \overrightarrow{\sigma })$, ultimately reflecting larger H_d_ and H_f_ values for Sample B even at the same current injection. In addition, the presence of relatively higher resistance in W buffer layer may be associated with the particularly enhanced Hf component of Sample B. The recent theoretical work 28 has suggested that the field-like torque can significantly be affected by the current flowing inside an FM layer with a 2D Rashba model or SHE model. In this sense, the W buffer layer that can absorb more boron and oxygen atoms during growth or annealing possibly creates a higher resistance than that of the Ta layer (See Supplementary Information S9), thus providing the remarkably enhanced Hf component in Sample B. The values of Hdand Hf reveal a strong θ dependence, as evident in the above equation and the empirical results of Fig. 4. However, questions remain regarding why there is an abnormal angular dependence of both components over the full θ range. In addition, the experimental observations cannot be completely described by either the SHE or Rashba model. The unclear θ-dependent phenomena observed in the full θ range are likely linked to a combination of two apparent dynamics, along with unknown additional structural contributions. Recent theoretical works^12,14,15,29^ addressed the θ-dependent behaviors of spin-orbit torque without identifying their origins. For example, the bulk SHE-based theories combined with the Boltzmann equation and a simpler drift-diffusion approach^30^ predicted no angular dependence of either Hf or Hd. On the other hand, the interfacial Rashba-based theories in a strong Rashba effect regime (compared to the exchange coupling strength^31^) showed a strong angular dependence of Hd through the anisotropy of the spin relaxation. However, Hf remains almost constant even after introduction of spin relaxation anisotropy. Thus, the θ dependence of diverse Hd and Hf reported by numerous studies^12,14,15,29^ (including our work) showed less agreement with the currently available theoretical models. Thus, attaining a firm understanding of the physical nature of these systems is still a major challenge that must be addressed to extend their use. Overall, a clearer model for the origin of current-induced effective fields observed in the samples must be developed.

**Figure 5:**
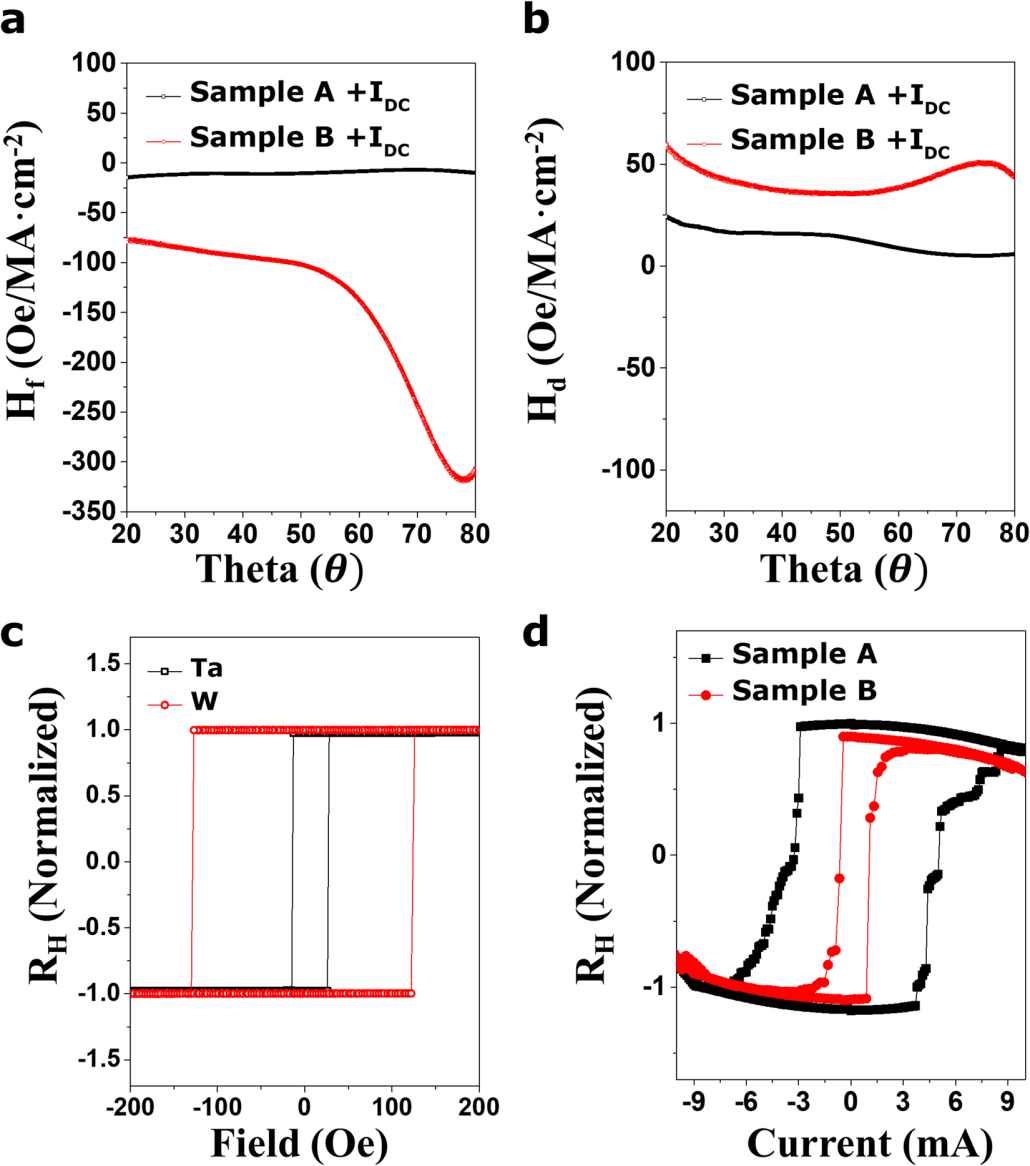
Comparison of *H*_*d*_ and *H*_*f*_ components and current-induced switching behavior for Samples A and B. (**a**) *H*_*f*_ and (**b**) H_*d*_ versus θ are plotted upon positive injection of +0.5 mA for Sample A (black lines) and B (red lines). Samples A and B always display relatively dominant *H*_*f*_ and H_*d*_ trends in the magnitude over the entire θ range, respectively. That is, the dissimilarity in the effective field characteristics of Samples A and B implies that the natures of the Ta and W HM layers are also inherently dissimilar. The clear enhancement in H_d_ for Sample B indicates the improved spin Hall angle, especially in Sample B (W-based sample). (**c**) The anomalous Hall resistance with out-of-plane field for Sample A and B. (**d**) Current switching with the external field of 50 Oe along the current direction for sample A and B. The normalized Hall resistance (R_H_) is plotted with the injected current for sample A (Black line) and sample B (Red line). The lower switching current is clearly observed for sample B case.

Figure 5 shows a plot the H_f_ (Fig. 5a) and H_d_ (Fig. 5b) of Samples A (black line) and B (red line) as a function of θ to emphasize the difference in magnitude of the current-induced effective fields. Experiments showed that the magnitude of H_d_ and H_f_ for Sample B was about 10 times (H_f_) and 2 times (H_d_) larger over the whole θ region. This increase in H_d_ and H_f_ magnitude for Sample B can be ascribed to the enhanced bulk-SHE or the current in an FM layer, as explained in Fig. 4 section. Given the important features of H_d_, the spin Hall angles (SHA, θ_SH_) for Samples A and B are calculated by ${H}_{d}=-\,\frac{\hslash }{2e}\cdot \frac{{J}_{HM}}{{\mu }_{0}{M}_{s}{d}_{FM}}\cdot {\theta }_{SH}$, here, $\hslash$ is the reduced Planck constant: $1.054\times {10}^{-34}\,J\cdot s$, e is the elementary charge: 1.602 × 10^−19^ *C*, J_HM_ is the current density through the HM: 5 × 10^10^ *A*/*m*^−2^, μ_0_ is the permeability of free space: $1.256\times {10}^{-6}\,N\cdot {{\rm{A}}}^{-2}$, M_s_ is the saturation magnetization: 1.203 × 10^6^*A*/*m* for Sample A and 1.259 × 10^6^ *A*/*m* for Sample B, and d_FM_ is the effective magnetic layer thickness corrected by considering magnetic dead layers (see Supplementary Information 7): 0.8 × 10^−9^ m for both samples. The calculated SHAs for Samples A and B are given in Figure S6. The determined SHA values (${\rm{Ta}} \sim -\,0.1$, ${\rm{W}} \sim -\,0.3$) are comparable with those in previous papers^20^. The enhancement of SHA arising from the β-phase W is clearly observed as expected. This feature can also be confirmed in the current switching behaviors for both samples. As seen in Fig. 5d, both samples with the identical FM thicknesses exhibit different switching currents: $\sim 3\times {10}^{7}\,{\rm{A}}/{{\rm{cm}}}^{-2}$ for sample A, $\sim 1\times {10}^{7}\,A/c{m}^{-2}$ for sample B. These switching current values are approximately well-matched with the relative magnitudes of H_d_ values observed from both samples (2 times larger in Sample B) because the current-induced magnetization switching is dominantly operated by the damping-like component^1^. However, the θ–dependent SHAs are still unclear. As seen in Fig. 5d, both samples with the identical FM thicknesses exhibit different switching currents: $\sim 3\times {10}^{7}\,{\rm{A}}/{{\rm{cm}}}^{-2}$ for sample A and $\sim 1\times {10}^{7}\,A/c{m}^{-2}$ for sample B. These switching current values are approximately well-matched with the relative magnitudes of H_d_ values observed from both samples (2 times larger in Sample B) because the current-induced magnetization switching is dominantly operated by the damping-like component. Since the intrinsic SHA value in the bulk HM does not rely on θ, the transparency of spin current injected into the FM layer at the HM/FM interface might be a possible origin of the variation in effective SHA (${{\rm{\theta }}}_{{\rm{SH}}}^{eff}$). To give rough estimates for the strong θ-dependent SHA, a brief interfacial spin-dependent scattering concept in a particular high θ regime is provided as proof of the possible nature. Zhang *et al*.^32^ have addressed that the interfacial transparency of spin current can be associated with the spin-mixing conductance, which is a function of magnetic damping constant. In addition, W. Kim *et al*.^33^ reported the angle-dependent magnetic damping constant possibly induced by the spin pumping effect. This paper pointed out the decrease in damping constant as the θ increased. Thus, the decreased damping constant can reduce the transparency of spin current at the interface when the following equation is used

8 $${\rm{T}}=\frac{{{\rm{G}}}_{\uparrow \downarrow }\,\tanh (\frac{{d}_{HM}}{2\lambda })}{{G}_{\uparrow \downarrow }\,\coth (\frac{{d}_{HM}}{\lambda })+\frac{{\sigma }_{HM}}{\lambda }\frac{{h}_{HM}}{2{e}^{2}}},$$

where ${{\rm{G}}}_{\uparrow \downarrow }$ is the spin-mixing conductance, λ is the spin diffusion length, σ_HM_ is the conductivity of heavy metals, h_HM_ is Planck’s constant and d_HM_ is the thickness of heavy metals. This models may explain the reduction in the effective SHA and the corresponding H_d_ as the θ increases. In addition, using Zhang’s initial transparency concept, another feasible scenario for the transparency could be as follows: if the injected spin current pointing along the y-axis exchanges with a total angular momentum ($\overrightarrow{{\rm{J}}}$) in d orbitals of an adjacent FM, the spin current transparency at the interface is also affected by the θ of FM when the θ-dependent barrier height is present at the interface. In this scenario, the H_*d*_ in a parallel scheme is not relevant to the θ dependence since the spin current injected along the y-axis is always perpendicular to the magnetization direction in a parallel scheme. The corresponding exchange interaction is equal for all magnetization polar angles. However, the H_*f*_ in a perpendicular scheme also has θ dependence due to the presence of $-\,{{\rm{J}}}_{{\rm{ex}}}\overrightarrow{M}\cdot \overrightarrow{\sigma }$, where $\overrightarrow{{\rm{\sigma }}}$ is directed along the y-axis. Thus, since movement toward the high θ regime allows the magnetization direction to primarily be close to the y-axis, the electron in the spin current will experience fewer scattering events due to the further reduced energy barrier. Such an event results in a spin current with large transparency, which facilitates injection of spin current. As a result, the H_f_is enhanced with an increase in θ, as seen in the high θ region of Fig. 4a,c. The angular dependence of H_f_ in the high θ region can be expressed by the transparency concept. However, the abnormal decrease in higher θ ($> 75^\circ$) and the behavior in the middle range ${\rm{\theta }}\,(20^\circ \sim 60^\circ )$ of both samples still remains a challenge so that a new physics beyond the currently available models should be established.

## Conclusions

In summary, in-plane DC measurements of spin-orbit torque components in Ta- and W-based CoFeB/MgO frames are employed as an independent analysis tool to examine the in-plane current-induced magnetization switching. The observed spin-orbit torque components for the two frames reveal a strong dependence on the magnetization of the polar angles. In particular, relatively larger Rashba and spin Hall dynamics are the dominant contributions to Samples A and B, respectively. Identifying the underlying nature of these phenomena remains a key challenge toward extending the use of these materials. One possible nature is the interfacial spin-dependent scattering arising from the exchange interactions between the angular momentum of d electrons in the CoFeB layer and the spin state of conduction electrons in the heavy metal layer. We anticipate that the study of this simple DC approach will open a suitable path to explore new physical phenomena and provide low power and high speed spin-orbit torque-based spintronic devices.

## Methods

The stacks used in this work were deposited on thermally oxidized Si substrates utilizing magnetron sputtering with a base pressure <2 × 10^−8^ Torr at room temperature. Species were as follows: [Si/SiO_2_(200)] substrate/heavy metals (5)/Co_20_Fe_60_*B*_20_ (t_CFB_)/MgO (1)/Ta (2), where the numbers in parentheses refer to the layer thickness in nanometers, and the heavy metals are Ta (Sample A) and W (Sample B). To promote perpendicular magnetic anisotropy (PMA) features, a post-annealing process was carried out at 250 °C (Sample A) and 300 °C (Sample B) for 1 hour under vacuum conditions below ~1 × 10^−6^ Torr with a 3 Tesla perpendicular magnetic field for all samples investigated here. The deposited stacks were spin-coated with AZ5214E image reversal photoresist and patterned into 10 μm width Hall bars by photolithography and Ar ion milling. Acetone was used to lift off the photo resist. Oxygen plasma etching was carried out for 2 minutes with 50 Watt RF power to remove residual photoresist hardened during the ion-milling process. The Hall channel contacts were defined by photolithography followed by the deposition of W (50 nm) and were connected to the Hall bars. Devices were wire-bonded to the sample holder using indium balls and were installed in a home-made electrical probing system with a ~1 Tesla electromagnet using a Keithley 236 source measure unit and Hewlett Packard 34401A multi-meter devices^34^.

## Electronic supplementary material

Supplementary Information
