# Supplementary material for: In-plane direct current probing for spin orbit torque-driven effective fields in perpendicularly magnetized heavy metal/ferromagnet/oxide frames
Source: Sci Rep. 2018 Jul 23;8:11065. doi: 10.1038/s41598-018-29397-4 (PMC6056570; doi:10.1038/s41598-018-29397-4)
Supplement: Supplementary file 1 — Supplementary Information [file 41598_2018_29397_MOESM1_ESM.docx]

SUPPLEMENTARY INFORMATION

Correspondence and requests for materials should be addressed to J.H ([jphong@hanyang.ac.kr](mailto:jphong@hanyang.ac.kr))

**In-plane direct current probing for spin orbit torque-driven effective fields in perpendicularly magnetized heavy metal/ferromagnet/oxide frames.**

Seungmo Yang^1^, Jinhyung Choi^1^, Wonsub Shin^1^, Kapsoo Yoon^1^, Jungyup Yang^2^, and JinPyo Hong^1,*^.

**1. Equations used in our approaches**

1. Determination of θ_0_ and θ values at each field.

To separate the total observed $R_{H}$ into a pristine Hall signal ($R_{H0}$) upon zero current and a variation in the Hall resistance ($\Delta R_{H}$) upon the injection of $I_{dc}$ with a certain magnitude, the $R_{H}$ of Equation (1) in the main article was approximated using a Taylor expansion only to the first order as follows:

$R_{H}\left( I_{dc} \right)\cong R_{H}\left( I_{dc}=0 \right)+I_{dc}\cdot{\frac{dR_{H}}{dI}}_{I=0}\equiv R_{H0}+\Delta R_{H}$ (S1)

Here, the Hall resistance in the absence of $I_{dc}$ is denoted by $R_{H0}\equiv R_{H}\left( I_{dc}=0 \right)$, and the first Taylor-expanded term is $I_{\mathrm{dc}}\cdot{\frac{\mathrm{dR}_{H}}{dI}}_{I=0}\equiv\Delta R_{H}$. The $R_{H0}$ and $\Delta R_{H}$ terms after the Taylor-expansion can be given by

$R_{H0}=R_{AHE}\cdot\cos\theta_{0}+R_{PHE}\cdot\sin^{2} \theta_{0}\cdot\sin2\varphi_{0}$ (S2)

$\Delta R_{H}=\left. I_{\mathrm{dc}}\cdot\frac{d}{dI}\left( R_{AHE}\cdot\cos\theta\left( I \right)+R_{PHE}\cdot\sin^{2} \theta\left( I \right)\cdot\sin2\varphi\left( I \right) \right) \right|_{I=0}$ (S3)

where $\theta_{0}$ and $\varphi_{0}$ are the polar and azimuthal angles for zero current, respectively. By taking the odd property of $\Delta R_{H}$ (equation (S3)) with respect to the${\pm I}_{\mathrm{dc}}$, the $R_{H0}$ and $\Delta R_{H}$ upon $\pm I_{dc}$can be obtained by

$R_{H0}=\frac{R_{H}\left( I_{dc} \right)+R_{H}\left( -I_{dc} \right)}{2}$ (S4)

$\Delta R_{H}=\frac{R_{H}\left( I_{dc} \right)-R_{H}\left( -I_{dc} \right)}{2}$ (S5)

Similarly, $\vec{H}_{ext}$-dependent characteristics were adopted to obtain the AHE-related term (first term) and the PHE-related term (second term) of Equation (S2). For example, the ($\theta_{0}$,$\varphi_{0}$) at the positive $H_{\mathrm{ext}}$ becomes ($\theta_{0}+\pi$,$\varphi_{0}+\pi$) under a negative $H_{\mathrm{ext}}$ for both schemes. Thus, the$\cos\theta_{0}$ in the AHE-related term at $H_{\mathrm{ext}}$ also becomes $\cos(\theta_{0}+\pi)=-\cos\theta_{0}$ for a negative$H_{\mathrm{ext}}$, verifying the odd function property of the AHE-related term with respect to the $\vec{H}_{\mathrm{ext}}$. However, the two terms [$\sin^{2} \theta_{0}$,$\sin2\varphi_{0}$] of the PHE-related term become [$\sin^{2} (\theta_{0}+\pi)$,$\sin2(\varphi_{0}+\pi)$] = [$\sin^{2} \theta_{0}$, $\sin2\varphi_{0}$] at the negative $H_{\mathrm{ext}}$, thereby providing an even function property with respect to the $\vec{H}_{\mathrm{ext}}$. Therefore, the utilization of the odd property in the AHE-related term with regard to $H_{\mathrm{ext}}$, the $R_{\mathrm{AHE}}\cdot\cos\theta_{0}$ and $R_{\mathrm{PHE}}\cdot\sin^{2} \theta_{0}\sin2\varphi_{0}$ of Equation (S2) upon the injection of $\pm H_{\mathrm{ext}}$ can be expressed by

$R_{\mathrm{AHE}}\cdot\cos\theta_{0}=\frac{R_{H0}(H_{ext})-R_{H0}\left( -H_{ext} \right)}{2}$ (S6)

$R_{PHE}\cdot\sin^{2} \theta_{0}\cdot\sin2\varphi_{0}=\frac{R_{H0}\left( H_{ext} \right)+R_{H0}\left( -H_{ext} \right)}{2}$ (S7)

Furthermore, the similar behavior of the AHE-related term in Equation (1) in the main article also determines the separation of two AHE- and PHE-associated terms with respect to $\pm H_{\mathrm{ext}}$ in the presence of$I_{dc}$. The resulting expressions are as follows:

$R_{\mathrm{AHE}}\cdot\cos\theta=\frac{R_{H}\left( H_{ext} \right)-R_{H}\left( -H_{ext} \right)}{2}$ (S8)

$R_{PHE}\cdot\sin^{2} \theta\cdot\sin2\varphi=\frac{R_{H}\left( H_{ext} \right)+R_{H}\left( -H_{ext} \right)}{2}$ (S9)

2. Determination of H_d_ and H_f_ from the θ_0_ and θ values

The total energy of the system is given by $E=-K_{\mathrm{eff}}\cos^{2} \theta-\vec{M}\cdot\vec{H}_{tot}$, where  $\vec{H}_{\mathrm{tot}}=\vec{H}_{ext}+\vec{H}_{d}+\vec{H}_{f}$; $\vec{H}_{ext}=H_{ext}(\sin\theta_{H}\cos\varphi_{H},\sin\theta_{H}\sin\varphi_{H},\cos\theta_{H})$ and the in-plane anisotropy was assumed to be negligible. The initial position ($\theta_{0},\varphi_{0}$) of the magnetization without the current ($\vec{H}_{tot}=\vec{H}_{ext}$)can be determined as follows,

$$\frac{\partial E}{\partial\theta}=0=K_{\mathrm{eff}}\sin2\theta_{0}-M_{s}H_{ext}(\cos\theta_{0}\cos\varphi_{0}\sin\theta_{H}\cos\varphi_{H}+\cos\theta_{0}\sin\varphi_{0}\sin\theta_{H}\sin\varphi_{H}-\sin\theta_{0}\cos\theta_{H})$$

$$\frac{\partial E}{\partial\varphi}=0=-M_{s}H\sin\theta_{0}\sin\theta_{H}\sin\left( \varphi_{H}-\varphi_{0} \right)$$

Then, the change in the position of the magnetization by the variation of $\vec{H}_{tot}$ generated by the presence of current-induced effective fields ($\vec{H}_{f}$ and $\vec{H}_{d}$) is expressed as

$$\Delta\theta=\frac{\partial\theta}{\partial H_{x}}\Delta H_{x}+\frac{\partial\theta}{\partial H_{y}}\Delta H_{y}+\frac{\partial\theta}{\partial H_{z}}\Delta H_{z}$$

$$\Delta\varphi=\frac{\partial\varphi}{\partial H_{x}}\Delta H_{x}+\frac{\partial\varphi}{\partial H_{y}}\Delta H_{y}+\frac{\partial\varphi}{\partial H_{z}}\Delta H_{z}$$

$$\Delta\theta=\frac{\left[ \cos\theta_{0}\left( \Delta H_{x}\cos\varphi_{H}+\Delta H_{y}\sin\varphi_{H} \right)-\sin\theta_{0}\Delta H_{z} \right]}{H_{k}\cos2\theta_{0}+H\cos(\theta_{H}-\theta_{0})}$$

$$\Delta\varphi=\frac{-\Delta H_{x}\sin\varphi_{H}+\Delta H_{y}\cos\varphi_{H}}{H\sin\theta_{H}}$$

1. Parallel measurement configuration case: H ∕∕ I

$$\vec{H}_{ext}=H_{ext}\left( \sin\theta_{H}\cos\varphi_{H},0,\cos\theta_{H} \right)$$

$$\Delta H_{x}=-H_{d}\cos\theta_{0}$$

$$\Delta H_{y}=H_{f}$$

$$\Delta H_{z}=H_{d}\sin\theta_{0}$$

$\boldsymbol{H}_{\boldsymbol{d}}\boldsymbol{=-\Delta\theta(}\boldsymbol{H}_{\boldsymbol{k}}\cos\boldsymbol{2}\boldsymbol{\theta}_{\boldsymbol{0}}\boldsymbol{+}\boldsymbol{H}_{\boldsymbol{ext}}\cos\left( \boldsymbol{\theta}_{\boldsymbol{H}}\boldsymbol{-}\boldsymbol{\theta}_{\boldsymbol{0}} \right)$

$$\boldsymbol{H}_{\boldsymbol{f}}\boldsymbol{=\Delta\varphi(H}\sin\boldsymbol{\theta}_{\boldsymbol{H}}\boldsymbol{)}$$

(2) Perpendicular measurement configuration case: H ⊥ I

$$\vec{H}_{ext}=H_{ext}\left( 0,\sin\theta_{H}\sin\varphi_{H},\cos\theta_{H} \right)$$

$$\Delta H_{x}=-H_{d}$$

$$\Delta H_{y}=H_{f}$$

$$\Delta H_{z}=0$$

$$\boldsymbol{H}_{\boldsymbol{f}}\boldsymbol{=[\Delta\theta(}\boldsymbol{H}_{\boldsymbol{k}}\cos\boldsymbol{2}\boldsymbol{\theta}_{\boldsymbol{0}}\boldsymbol{+}\boldsymbol{H}_{\boldsymbol{ext}}\cos\left( \boldsymbol{\theta}_{\boldsymbol{H}}\boldsymbol{-}\boldsymbol{\theta}_{\boldsymbol{0}} \right)\boldsymbol{]/}\cos\boldsymbol{\theta}_{\boldsymbol{0}}$$

$$\boldsymbol{H}_{\boldsymbol{d}}\boldsymbol{=}\boldsymbol{\Delta}\boldsymbol{\varphi}\left( \boldsymbol{H}\sin\boldsymbol{\theta}_{\boldsymbol{H}} \right)$$

3. the equivalence of the suggested approach with LLGS equation

LLGS equation is written as following.

$$\frac{\partial\vec{m}}{\partial t}=-\left| \gamma\right|\vec{m}\times\vec{H_{ext}}+\alpha\left( \vec{m}\times\frac{d\vec{m}}{dt} \right)+\left| \gamma\right|H_{d}\vec{m}\times\left( \vec{m}\times\hat{\sigma} \right)+\left| \gamma\right|H_{f}\vec{m}\times\hat{\sigma}$$

Due to the presence of magnetic damping term (the second term on the right hand), the magnetic moment tends to align along the direction of minimum energy state, which is parallel to the total effective field. Thus, LLGS equation can be rewritten by

$$\frac{\partial\vec{m}}{\partial t}=-\left| \gamma\right|\vec{m}\times[\vec{H_{ext}}+H_{d}\left( \vec{m}\times\hat{\sigma} \right)+H_{f}\hat{\sigma}]+\alpha\left( \vec{m}\times\frac{d\vec{m}}{dt} \right)$$

,where the total effective field  $\vec{H_{tot}}$ is $[\vec{H_{ext}}+H_{d}\left( \vec{m}\times\hat{\sigma} \right)+H_{f}\hat{\sigma}]$, $\vec{H_{ext}}$ includes the external magnetic field and the anisotropy field.

Then, at equilibrium ($\frac{\partial\vec{m}}{\partial t}=0$), the static LLGS equation becomes to

$$0=-\left| \gamma\right|\vec{m}\times[\vec{H_{ext}}+H_{d}\left( \vec{m}\times\hat{\sigma} \right)+H_{f}\hat{\sigma}]$$

, which means that $\vec{m}$ is parallel to the total effective field ($\vec{H_{ext}}+H_{d}\left( \vec{m}\times\hat{\sigma} \right)+H_{f}\hat{\sigma}$).

This approach is actually equal to the angle deviation about the total magnetic energy with the added two effective fields ($\vec{H_{d}} \& \vec{H_{f}}$). However, we cannot directly solve static LLGS equation because of the presence of two unknown values ($H_{d} \& H_{f}$).

In this reason, X, Qiu et al. [Sci. Rep. 4, 4491 (2014)] solved the above derivation with two unknown value using parameter fitting method using with 2f harmonic signals, which is a quite complicate task.

However, the angle partial deviation of the total magnetic energy at particular geometries (parallel & perpendicular configuration) can split two unknown values ($H_{d} \& H_{f}$) into two individual equations, which makes $H_{d} \& H_{f}$ values relatively easily obtained.

**2. Various Hall signals observed from Sample B**

**Figure S2.**

**
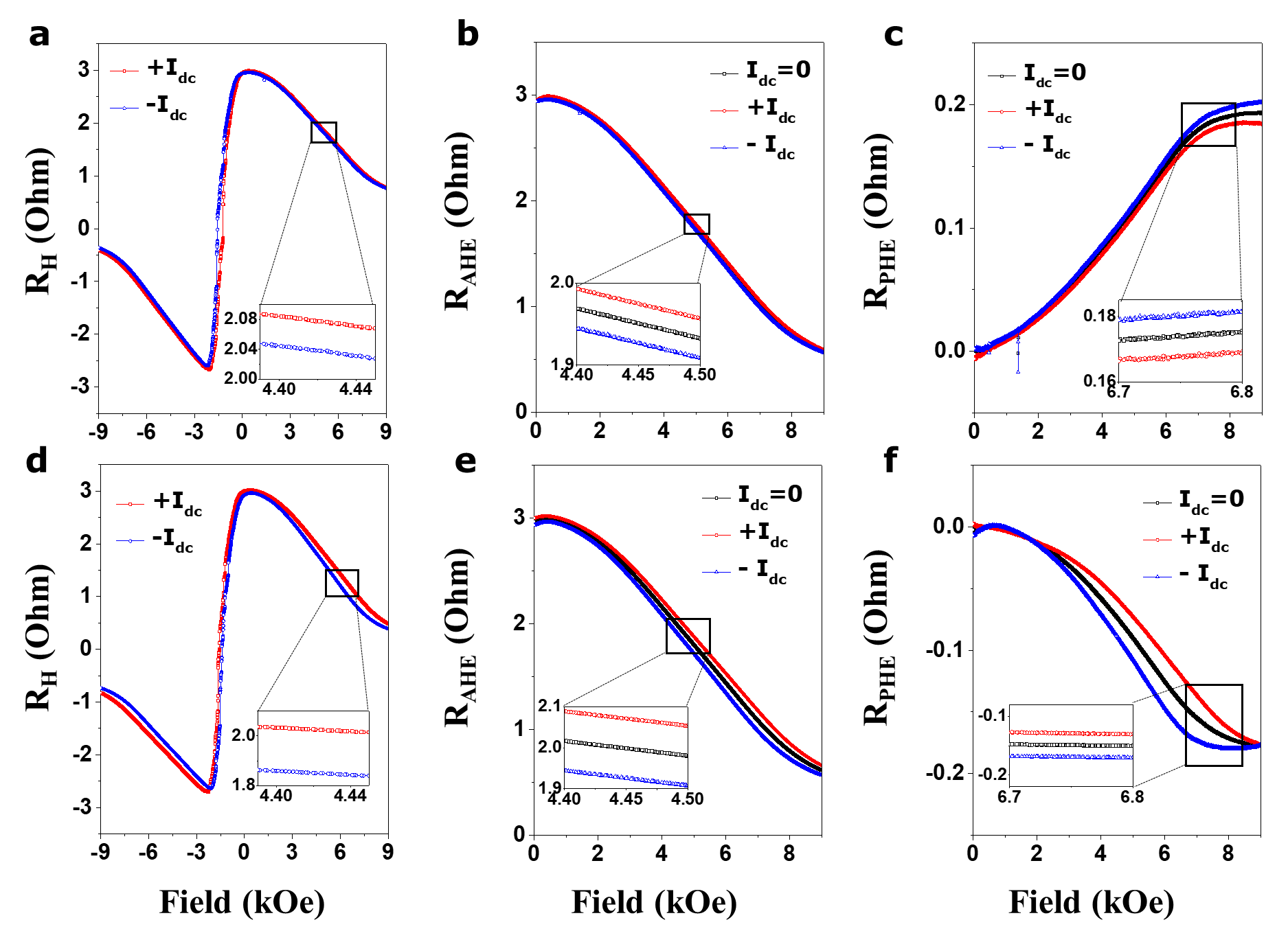
**

**Fig, S2.** DC source-induced magnetic features for the W-based sample [Sample B]. **(a)** $R_{H}$ versus $H_{\mathrm{ext}}$ parallel to the positive (black line) and negative (red line) current $I_{dc}$ for $I_{dc}=0.5 mA$. **(b, c)** Separated $R_{AHE}$ and $R_{\mathrm{PHE}}$ as a function of $H_{\mathrm{ext}}$ parallel to zero-current (black line), +0.5 mA (red line) and -0.5 mA (blue line). **(d)** $R_{H}$ versus $H_{ext}$ perpendicular to the positive (black line) and negative (red line) current $I_{dc}$ for $I_{dc}=0.5 mA$. **(e, f)** similar plots of $R_{AHE}$ and $R_{PHE}$ versus $H_{ext}$ perpendicular to three different currents, $I_{dc}=0$ (black line), +0.5 $\mathrm{mA}$ (red line) and -0.5 $\mathrm{mA}$ (blue line). Insets: the magnified features of the main graphs reflect the difference in the main curves at ${\pm I}_{dc}$ current.

**3. Magnetic features of Samples A and B**

**Figure S3.**

**
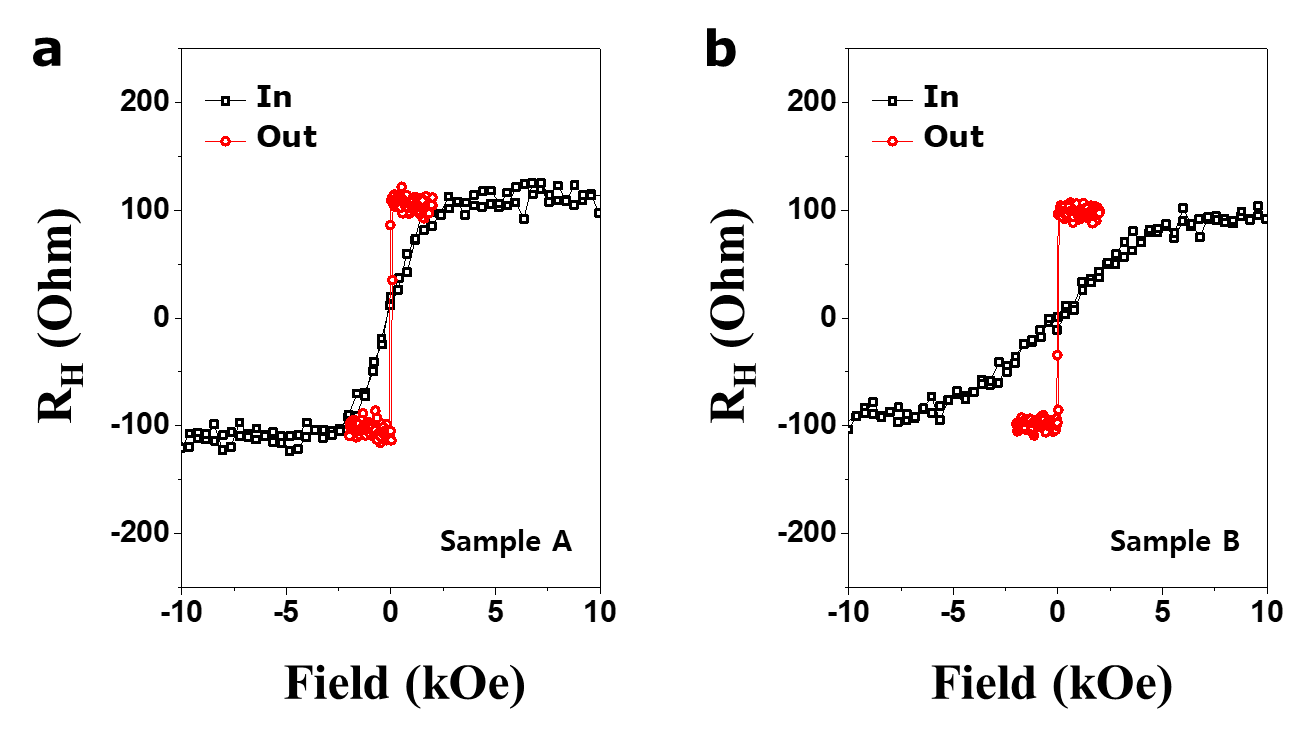
**

**Fig. S3.** Magnetic hysteresis loops for Samples A and B. In-plane (black line) and out-of-plane (red line) magnetic hysteresis loops monitored by the VSM system for **(a)** Sample A and **(b)** Sample B. The $H_{\mathrm{an}}$values of Samples A and B are ~3.4 kOe and ~ 5.2 kOe, respectively.

**4. XRD analysis for Ta and W HM layers**

**Figure S4.**

**
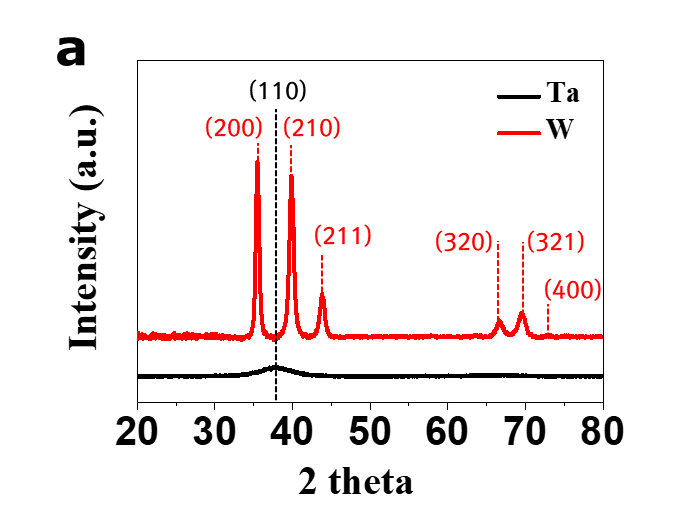
**

**Fig. S4.** Crystalline analysis of Ta and W layers grown on SiO_2._ **(a)** X-Ray diffraction patterns for the Ta and W heavy metal layers serving as buffer layers for Samples A and B. Samples A and B exhibit clear BCC (black line) and A15 (red line) crystalline features, respectively.

**5. Additional contribution in low theta regions**

**Figure S5.**


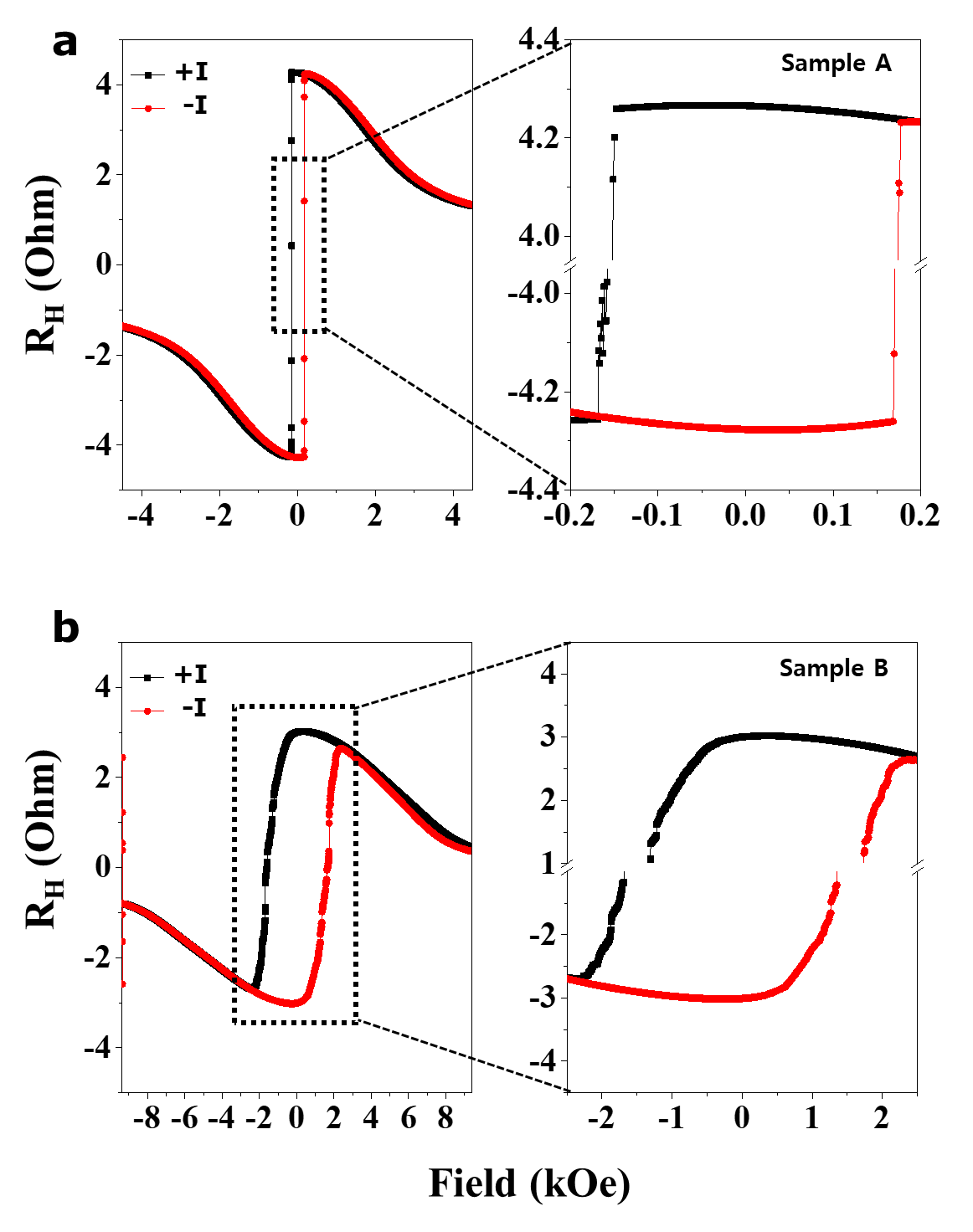


**Fig. S5.** instability issues within coercivity fields**.** Anomalous $R_{H}$ (Top) as a function of H applied along the positive (+0.5 mA, black line) and negative current (-0.5 mA, red line) for Samples A and B **(a, b)**, respectively. Note that the magnified features (Bottom) in the low $\theta$ regions for both samples clearly reveal the domain wall pinning effect (dashed squares) that can additionally contribute to the total effective magnetic field. Therefore, this low region is excluded in our approach due to the difficulty of the analysis.

**6. Spin Hall angles for Samples A and B**

**Figure S6.**


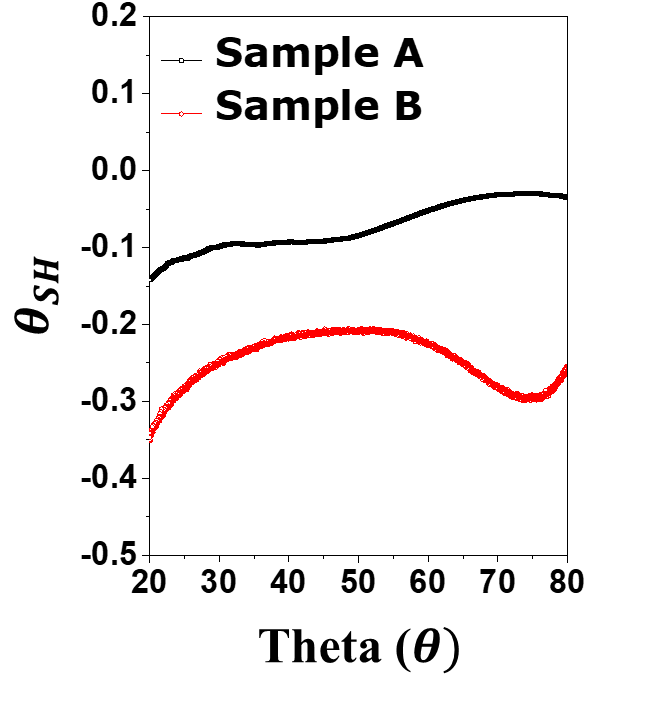


**Fig. S6.** Spin Hall angles ($\theta_{\mathrm{SH}}$) for Samples A and B**.** The spin Hall angle (SHA, $\theta_{\mathrm{SH}}$) is evaluated from the $H_{d}$ values for Samples A and B. As seen, Sample B (W) shows enhanced SHAs (about 2 times larger than that of Sample A), along with a strong dependence on $\theta$.

**7. Magnetic dead layer and saturation magnetization fitting**

**Figure S7.**


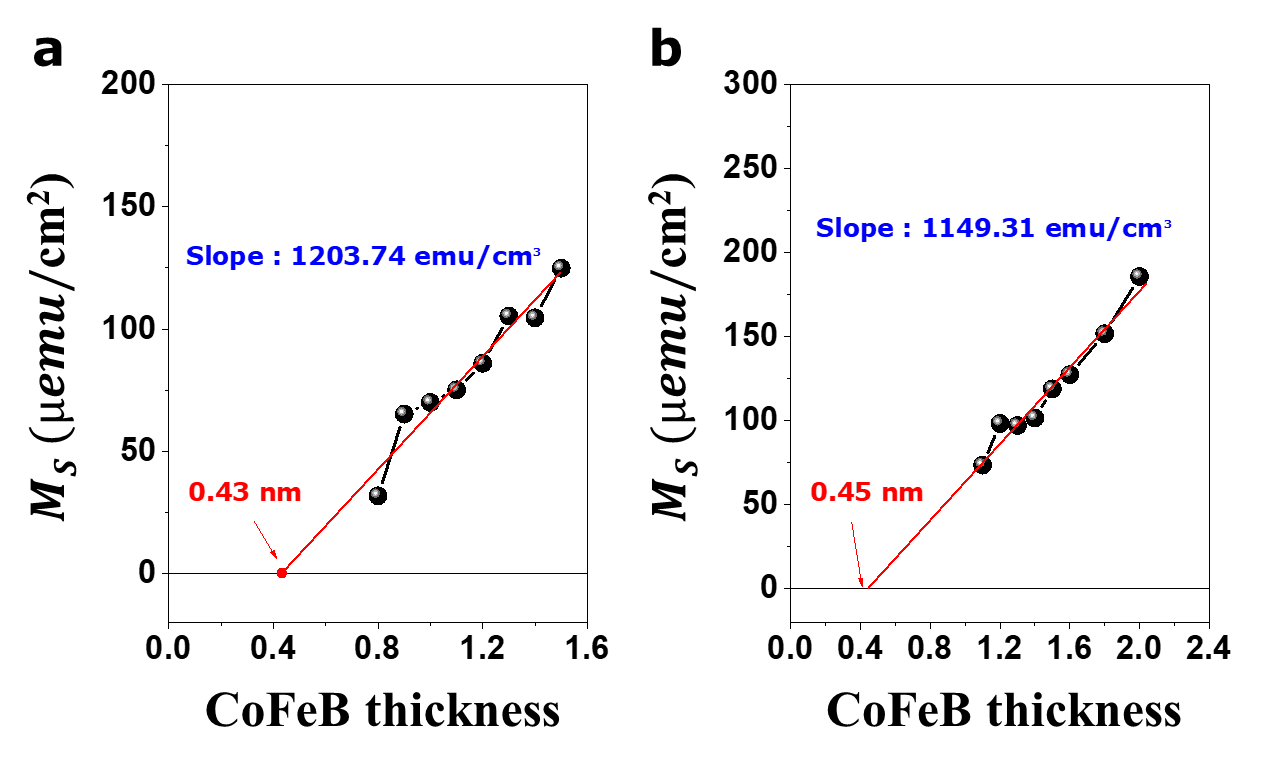


**Fig. S7.** Saturation magnetization and dead layer fits of Ta- and W-buffer samples. The areal saturation magnetization ($M_{s}/Area$) is plotted as a function of CoFeB layer thickness for **(a)** Ta buffer stacks [SiO_2_ / Ta (5) / CoFeB (t_CFB_) / MgO (1) / Ta (2) after 250 ℃ annealing] and **(b)** W buffer stacks [SiO_2_ / W (5) / CoFeB (t_CFB_) / MgO (1) / Ta (2) after 350 ℃ annealing]. Linear fitted lines (red lines) in both graphs indicate the saturation magnetization.

**8. Possible reason for the presence of high resistivity in W layer.**

**Figure S8.**


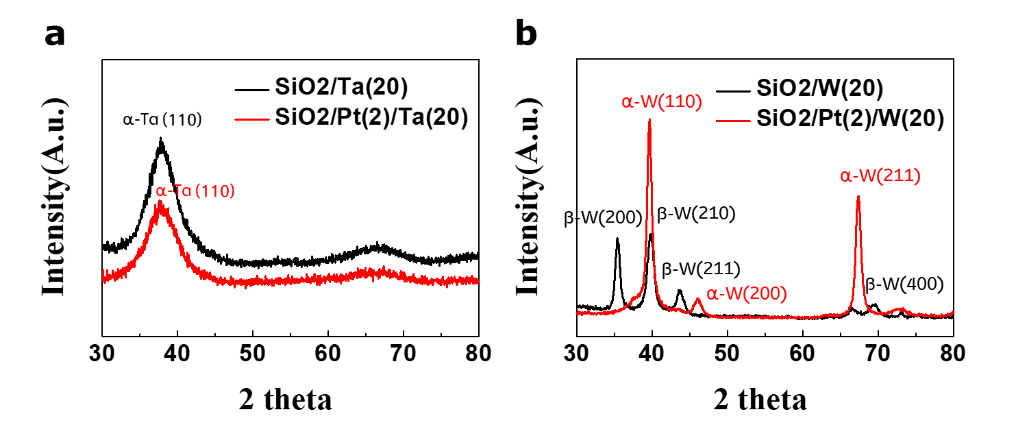


**Fig. S8.** Crystalline analysis of Ta and W layers grown on SiO_2_ and Pt layer. **(a)** X-Ray diffraction patterns for the Ta and **(b)** for the W on SiO_2_ (Black line) and Pt layer (Red line). W layer only exhibits the phase change using Pt buffer layer, which possibly prevents oxygen atoms in SiO_2_ layer to diffuse into W layer. However, Ta layer presents the identical structure regardless of the presence of Pt layer, indicating $\alpha$-phase Ta is robust enough to maintain the structure even with the adjacent oxygen atoms.

**9. Calculations of the current-induced Oersted.**

**Figure S9.**


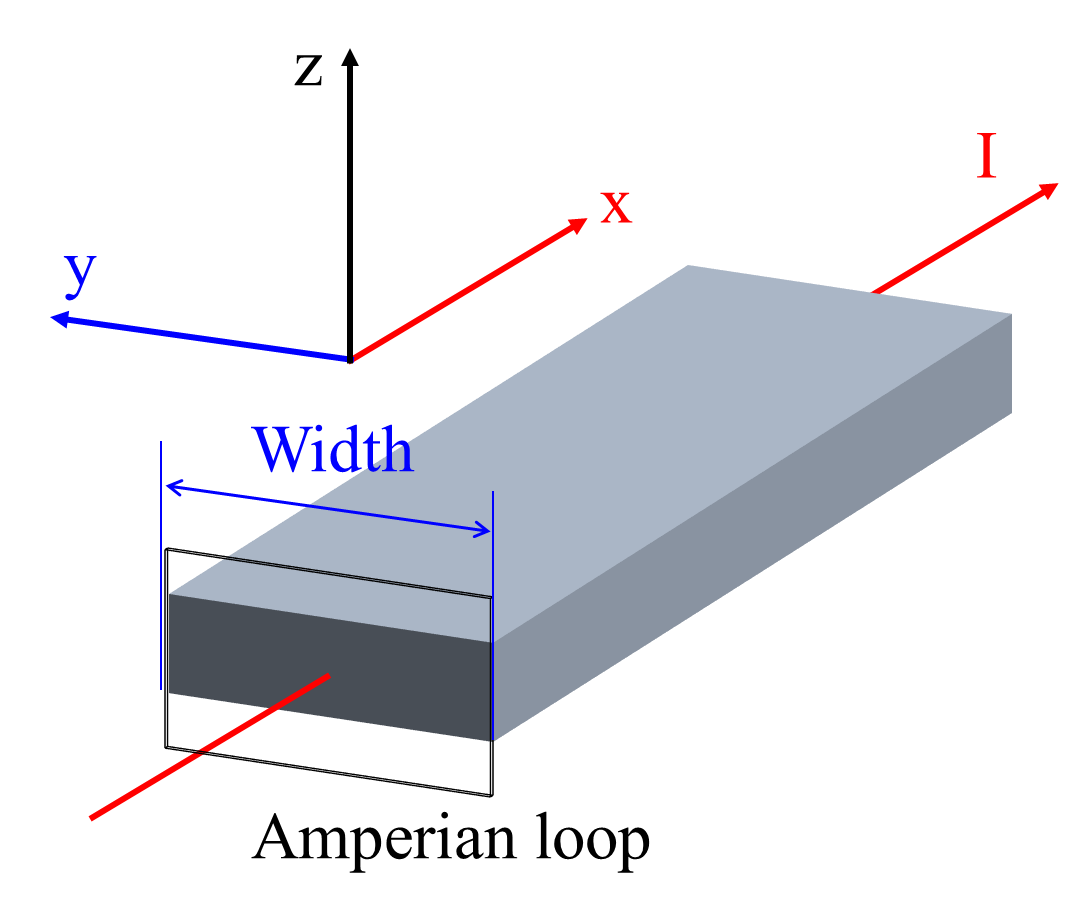


**Fig. S9.** Determination of Oersted field by assuming an infinite conducting plate. When the Ampere’s law $\oint_{C} \vec{B}\cdot d\vec{l}=\mu_{0}I$, width ω and current density $\vec{J_{x}}=J_{x}\hat{x}$ are used, the Oersted field is given by $H=\frac{I}{2\omega}$. The Oersted field was 0.314159 Oe along the y-axis with $I=0.5 mA, \omega=10\mu m$.
